# Supplementary material for: The Evolution of Dietary Consumption in the Spanish Adult Population and Its Relationship with Environmental Sustainability
Source: Nutrients. 2024 Dec 20;16(24):4391. doi: 10.3390/nu16244391 (PMC11677257; doi:10.3390/nu16244391)
Supplement: Supplementary file 1 [file nutrients-16-04391-s001.zip › nutrients-3371864-supplementary.pdf]

Food group	title	Class	content
Dairy	Milk	GHG (g CO2-eq/g)	1.12166781
Dairy	Milk	LU (m2/g)	0.00166925
Dairy	Milk	EU (kJ/g)	2.914615385
Dairy	Milk	Acidification_potencial (g SO2-eq/g)	0.023163309
Dairy	Milk	Eutrophication_potencial (g PO4-eq/g)	0.013932804
Dairy	Yogurt	GHG (g CO2-eq/g)	1.41339
Dairy	Yogurt	LU (m2/g)	0.000522
Dairy	Yogurt	EU (kJ/g)	
Dairy	Yogurt	Acidification_potencial (g SO2-eq/g)	0.014665799
Dairy	Yogurt	Eutrophication_potencial (g PO4-eq/g)	6.31E-03
Dairy	Cheese	GHG (g CO2-eq/g)	11.491875
Dairy	Cheese	LU (m2/g)	
Dairy	Cheese	EU (kJ/g)	
Dairy	Cheese	Acidification_potencial (g SO2-eq/g)	0.131792857
Dairy	Cheese	Eutrophication_potencial (g PO4-eq/g)	
Dairy	Cream	GHG (g CO2-eq/g)	4.161
Dairy	Cream	LU (m2/g)	
Dairy	Cream	EU (kJ/g)	
Dairy	Cream	Acidification_potencial (g SO2-eq/g)	0.0504
Dairy	Cream	Eutrophication_potencial (g PO4-eq/g)	0.023653333
Eggs	Eggs	GHG (g CO2-eq/g)	3.025873016
Eggs	Eggs	LU (m2/g)	0.006450526
Eggs	Eggs	EU (kJ/g)	16.69210526
Eggs	Eggs	Acidification_potencial (g SO2-eq/g)	0.07210339
Eggs	Eggs	Eutrophication_potencial (g PO4-eq/g)	0.031575758
Fresh Produce	Andean blackberry	GHG (g CO2-eq/g)	0.17
Fresh Produce	Andean blackberry	LU (m2/g)	0.00115
Fresh Produce	Andean blackberry	EU (kJ/g)	
Fresh Produce	Andean blackberry	Acidification_potencial (g SO2-eq/g)	
Fresh Produce	Andean blackberry	Eutrophication_potencial (g PO4-eq/g)	
Fresh Produce	Apples	GHG (g CO2-eq/g)	0.102019048
Fresh Produce	Apples	LU (m2/g)	0.000220211

Fresh Produce	Apples	EU (kJ/g)	0.719666667
Fresh Produce	Apples	Acidification_potencial (g SO2-eq/g)	0.000807667
Fresh Produce	Apples	Eutrophication_potencial (g PO4-eq/g)	0.0003415
Fresh Produce	Asparagus	GHG (g CO2-eq/g)	0.676333333
Fresh Produce	Asparagus	LU (m2/g)	0.00118
Fresh Produce	Asparagus	EU (kJ/g)	1.18
Fresh Produce	Asparagus	Acidification_potencial (g SO2-eq/g)	
Fresh Produce	Asparagus	Eutrophication_potencial (g PO4-eq/g)	
Fresh Produce	Avocado	GHG (g CO2-eq/g)	0.166
Fresh Produce	Avocado	LU (m2/g)	0.00106
Fresh Produce	Avocado	EU (kJ/g)	
Fresh Produce	Avocado	Acidification_potencial (g SO2-eq/g)	
Fresh Produce	Avocado	Eutrophication_potencial (g PO4-eq/g)	
Fresh Produce	Banana	GHG (g CO2-eq/g)	0.236666667
Fresh Produce	Banana	LU (m2/g)	0.000307
Fresh Produce	Banana	EU (kJ/g)	
Fresh Produce	Banana	Acidification_potencial (g SO2-eq/g)	
Fresh Produce	Banana	Eutrophication_potencial (g PO4-eq/g)	
Fresh Produce	Blueberries	GHG (g CO2-eq/g)	0.113
Fresh Produce	Blueberries	LU (m2/g)	
Fresh Produce	Blueberries	EU (kJ/g)	2.19
Fresh Produce	Blueberries	Acidification_potencial (g SO2-eq/g)	
Fresh Produce	Blueberries	Eutrophication_potencial (g PO4-eq/g)	
Fresh Produce	Cherry tomato	GHG (g CO2-eq/g)	0.870632911
Fresh Produce	Cherry tomato	LU (m2/g)	
Fresh Produce	Cherry tomato	EU (kJ/g)	16.07229114
Fresh Produce	Cherry tomato	Acidification_potencial (g SO2-eq/g)	0.006848101
Fresh Produce	Cherry tomato	Eutrophication_potencial (g PO4-eq/g)	0.002585443
Fresh Produce	Chicory	GHG (g CO2-eq/g)	0.132
Fresh Produce	Chicory	LU (m2/g)	0.000208
Fresh Produce	Chicory	EU (kJ/g)	
Fresh Produce	Chicory	Acidification_potencial (g SO2-eq/g)	0.000547
Fresh Produce	Chicory	Eutrophication_potencial (g PO4-eq/g)	0.00081

Fresh Produce	Chinese Pear	GHG (g CO2-eq/g)	0.1564
Fresh Produce	Chinese Pear	LU (m2/g)	0.000377
Fresh Produce	Chinese Pear	EU (kJ/g)	1.3254
Fresh Produce	Chinese Pear	Acidification_potencial (g SO2-eq/g)	
Fresh Produce	Chinese Pear	Eutrophication_potencial (g PO4-eq/g)	
Fresh Produce	Escarole	GHG (g CO2-eq/g)	0.0399
Fresh Produce	Escarole	LU (m2/g)	
Fresh Produce	Escarole	EU (kJ/g)	
Fresh Produce	Escarole	Acidification_potencial (g SO2-eq/g)	
Fresh Produce	Escarole	Eutrophication_potencial (g PO4-eq/g)	
Fresh Produce	Golden berry	GHG (g CO2-eq/g)	0.241
Fresh Produce	Golden berry	LU (m2/g)	0.000514
Fresh Produce	Golden berry	EU (kJ/g)	
Fresh Produce	Golden berry	Acidification_potencial (g SO2-eq/g)	
Fresh Produce	Golden berry	Eutrophication_potencial (g PO4-eq/g)	
Fresh Produce	Grapes	GHG (g CO2-eq/g)	0.31475
Fresh Produce	Grapes	LU (m2/g)	0.001102167
Fresh Produce	Grapes	EU (kJ/g)	
Fresh Produce	Grapes	Acidification_potencial (g SO2-eq/g)	0.002925
Fresh Produce	Grapes	Eutrophication_potencial (g PO4-eq/g)	0.015925
Fresh Produce	Kiwis	GHG (g CO2-eq/g)	0.819
Fresh Produce	Kiwis	LU (m2/g)	0.000478
Fresh Produce	Kiwis	EU (kJ/g)	
Fresh Produce	Kiwis	Acidification_potencial (g SO2-eq/g)	
Fresh Produce	Kiwis	Eutrophication_potencial (g PO4-eq/g)	
Fresh Produce	Leeks	GHG (g CO2-eq/g)	0.06895
Fresh Produce	Leeks	LU (m2/g)	0.000315
Fresh Produce	Leeks	EU (kJ/g)	
Fresh Produce	Leeks	Acidification_potencial (g SO2-eq/g)	0.0004165
Fresh Produce	Leeks	Eutrophication_potencial (g PO4-eq/g)	0.000684
Fresh Produce	Lemons	GHG (g CO2-eq/g)	0.0873
Fresh Produce	Lemons	LU (m2/g)	0.00000943
Fresh Produce	Lemons	EU (kJ/g)	2.65

Fresh Produce	Lemons	Acidification_potencial (g SO2-eq/g)	
Fresh Produce	Lemons	Eutrophication_potencial (g PO4-eq/g)	
Fresh Produce	Lettuce	GHG (g CO2-eq/g)	0.025
Fresh Produce	Lettuce	LU (m2/g)	
Fresh Produce	Lettuce	EU (kJ/g)	
Fresh Produce	Lettuce	Acidification_potencial (g SO2-eq/g)	
Fresh Produce	Lettuce	Eutrophication_potencial (g PO4-eq/g)	
Fresh Produce	Lulo	GHG (g CO2-eq/g)	0.384
Fresh Produce	Lulo	LU (m2/g)	0.00121
Fresh Produce	Lulo	EU (kJ/g)	
Fresh Produce	Lulo	Acidification_potencial (g SO2-eq/g)	
Fresh Produce	Lulo	Eutrophication_potencial (g PO4-eq/g)	
Fresh Produce	Mango	GHG (g CO2-eq/g)	0.052
Fresh Produce	Mango	LU (m2/g)	0.00118
Fresh Produce	Mango	EU (kJ/g)	
Fresh Produce	Mango	Acidification_potencial (g SO2-eq/g)	
Fresh Produce	Mango	Eutrophication_potencial (g PO4-eq/g)	
Fresh Produce	Mushrooms	GHG (g CO2-eq/g)	1.01
Fresh Produce	Mushrooms	LU (m2/g)	
Fresh Produce	Mushrooms	EU (kJ/g)	
Fresh Produce	Mushrooms	Acidification_potencial (g SO2-eq/g)	
Fresh Produce	Mushrooms	Eutrophication_potencial (g PO4-eq/g)	
Fresh Produce	Onions	GHG (g CO2-eq/g)	0.0506
Fresh Produce	Onions	LU (m2/g)	
Fresh Produce	Onions	EU (kJ/g)	0.397
Fresh Produce	Onions	Acidification_potencial (g SO2-eq/g)	
Fresh Produce	Onions	Eutrophication_potencial (g PO4-eq/g)	
Fresh Produce	Oranges	GHG (g CO2-eq/g)	0.160666667
Fresh Produce	Oranges	LU (m2/g)	0.00000643
Fresh Produce	Oranges	EU (kJ/g)	2.625
Fresh Produce	Oranges	Acidification_potencial (g SO2-eq/g)	
Fresh Produce	Oranges	Eutrophication_potencial (g PO4-eq/g)	
Fresh Produce	Passion fruit	GHG (g CO2-eq/g)	0.107

Fresh Produce	Passion fruit	LU (m2/g)	0.000581
Fresh Produce	Passion fruit	EU (kJ/g)	
Fresh Produce	Passion fruit	Acidification_potencial (g SO2-eq/g)	
Fresh Produce	Passion fruit	Eutrophication_potencial (g PO4-eq/g)	
Fresh Produce	Peaches	GHG (g CO2-eq/g)	0.095866667
Fresh Produce	Peaches	LU (m2/g)	0.000465333
Fresh Produce	Peaches	EU (kJ/g)	
Fresh Produce	Peaches	Acidification_potencial (g SO2-eq/g)	
Fresh Produce	Peaches	Eutrophication_potencial (g PO4-eq/g)	
Fresh Produce	Pear	GHG (g CO2-eq/g)	0.376
Fresh Produce	Pear	LU (m2/g)	0.000331
Fresh Produce	Pear	EU (kJ/g)	
Fresh Produce	Pear	Acidification_potencial (g SO2-eq/g)	0.00413
Fresh Produce	Pear	Eutrophication_potencial (g PO4-eq/g)	0.00126
Fresh Produce	Pepper	GHG (g CO2-eq/g)	0.9094
Fresh Produce	Pepper	LU (m2/g)	
Fresh Produce	Pepper	EU (kJ/g)	17.88
Fresh Produce	Pepper	Acidification_potencial (g SO2-eq/g)	0.006854
Fresh Produce	Pepper	Eutrophication_potencial (g PO4-eq/g)	0.003377333
Fresh Produce	Pineapple	GHG (g CO2-eq/g)	0.0976
Fresh Produce	Pineapple	LU (m2/g)	0.0000636
Fresh Produce	Pineapple	EU (kJ/g)	1.67
Fresh Produce	Pineapple	Acidification_potencial (g SO2-eq/g)	
Fresh Produce	Pineapple	Eutrophication_potencial (g PO4-eq/g)	0.00332
Fresh Produce	Potatoes	GHG (g CO2-eq/g)	0.153333333
Fresh Produce	Potatoes	LU (m2/g)	0.000335714
Fresh Produce	Potatoes	EU (kJ/g)	1.27
Fresh Produce	Potatoes	Acidification_potencial (g SO2-eq/g)	0.00135
Fresh Produce	Potatoes	Eutrophication_potencial (g PO4-eq/g)	0.00115
Fresh Produce	Raspberries	GHG (g CO2-eq/g)	0.337
Fresh Produce	Raspberries	LU (m2/g)	
Fresh Produce	Raspberries	EU (kJ/g)	6.6
Fresh Produce	Raspberries	Acidification_potencial (g SO2-eq/g)	

Fresh Produce	Raspberries	Eutrophication_potencial (g PO4-eq/g)	
Fresh Produce	Romaine Lettuce	GHG (g CO2-eq/g)	0.844333333
Fresh Produce	Romaine Lettuce	LU (m2/g)	
Fresh Produce	Romaine Lettuce	EU (kJ/g)	
Fresh Produce	Romaine Lettuce	Acidification_potencial (g SO2-eq/g)	
Fresh Produce	Romaine Lettuce	Eutrophication_potencial (g PO4-eq/g)	
Fresh Produce	Spinach	GHG (g CO2-eq/g)	2.3
Fresh Produce	Spinach	LU (m2/g)	
Fresh Produce	Spinach	EU (kJ/g)	
Fresh Produce	Spinach	Acidification_potencial (g SO2-eq/g)	
Fresh Produce	Spinach	Eutrophication_potencial (g PO4-eq/g)	
Fresh Produce	Strawberries	GHG (g CO2-eq/g)	0.6135
Fresh Produce	Strawberries	LU (m2/g)	0.00183
Fresh Produce	Strawberries	EU (kJ/g)	
Fresh Produce	Strawberries	Acidification_potencial (g SO2-eq/g)	0.00256
Fresh Produce	Strawberries	Eutrophication_potencial (g PO4-eq/g)	0.00038
Fresh Produce	Tomatoes	GHG (g CO2-eq/g)	0.202
Fresh Produce	Tomatoes	LU (m2/g)	0.0001077
Fresh Produce	Tomatoes	EU (kJ/g)	5.263333333
Fresh Produce	Tomatoes	Acidification_potencial (g SO2-eq/g)	0.000966667
Fresh Produce	Tomatoes	Eutrophication_potencial (g PO4-eq/g)	0.000493
Fresh Produce	Tree tomato	GHG (g CO2-eq/g)	0.263
Fresh Produce	Tree tomato	LU (m2/g)	0.000456
Fresh Produce	Tree tomato	EU (kJ/g)	
Fresh Produce	Tree tomato	Acidification_potencial (g SO2-eq/g)	
Fresh Produce	Tree tomato	Eutrophication_potencial (g PO4-eq/g)	
Fresh Produce	Zucchini	GHG (g CO2-eq/g)	1.214596774
Fresh Produce	Zucchini	LU (m2/g)	
Fresh Produce	Zucchini	EU (kJ/g)	22.42068548
Fresh Produce	Zucchini	Acidification_potencial (g SO2-eq/g)	0.010050806
Fresh Produce	Zucchini	Eutrophication_potencial (g PO4-eq/g)	0.005179839
Barley	Barley	GHG (g CO2-eq/g)	0.69724026
Barley	Barley	LU (m2/g)	0.009613636

Barley	Barley	EU (kJ/g)	
Barley	Barley	Acidification_potencial (g SO2-eq/g)	
Barley	Barley	Eutrophication_potencial (g PO4-eq/g)	
Maize	Maize	GHG (g CO2-eq/g)	0.416461538
Maize	Maize	LU (m2/g)	0.001351538
Maize	Maize	EU (kJ/g)	2.65
Maize	Maize	Acidification_potencial (g SO2-eq/g)	0.006401538
Maize	Maize	Eutrophication_potencial (g PO4-eq/g)	0.002160909
Oat	Oat	GHG (g CO2-eq/g)	0.77
Oat	Oat	LU (m2/g)	0.002214286
Oat	Oat	EU (kJ/g)	
Oat	Oat	Acidification_potencial (g SO2-eq/g)	
Oat	Oat	Eutrophication_potencial (g PO4-eq/g)	
Rice	Rice	GHG (g CO2-eq/g)	1.679333333
Rice	Rice	LU (m2/g)	0.001818462
Rice	Rice	EU (kJ/g)	7.847142857
Rice	Rice	Acidification_potencial (g SO2-eq/g)	0.01895
Rice	Rice	Eutrophication_potencial (g PO4-eq/g)	0.008633333
Wheat	Wheat	GHG (g CO2-eq/g)	0.712162162
Wheat	Wheat	LU (m2/g)	0.005450313
Wheat	Wheat	EU (kJ/g)	2.204375
Wheat	Wheat	Acidification_potencial (g SO2-eq/g)	0.009475556
Wheat	Wheat	Eutrophication_potencial (g PO4-eq/g)	0.004617571
Fish and seafood	Catfish	GHG (g CO2-eq/g)	21.9
Fish and seafood	Catfish	LU (m2/g)	
Fish and seafood	Catfish	EU (kJ/g)	
Fish and seafood	Catfish	Acidification_potencial (g SO2-eq/g)	
Fish and seafood	Catfish	Eutrophication_potencial (g PO4-eq/g)	
Fish and seafood	Mussels	GHG (g CO2-eq/g)	5.575
Fish and seafood	Mussels	LU (m2/g)	
Fish and seafood	Mussels	EU (kJ/g)	
Fish and seafood	Mussels	Acidification_potencial (g SO2-eq/g)	
Fish and seafood	Mussels	Eutrophication_potencial (g PO4-eq/g)	0.000107

Fish and seafood	Salmon	GHG (g CO2-eq/g)	5.178888889
Fish and seafood	Salmon	LU (m2/g)	
Fish and seafood	Salmon	EU (kJ/g)	
Fish and seafood	Salmon	Acidification_potencial (g SO2-eq/g)	
Fish and seafood	Salmon	Eutrophication_potencial (g PO4-eq/g)	5.60857E-05
Fish and seafood	Sea-bass	GHG (g CO2-eq/g)	18.77
Fish and seafood	Sea-bass	LU (m2/g)	
Fish and seafood	Sea-bass	EU (kJ/g)	
Fish and seafood	Sea-bass	Acidification_potencial (g SO2-eq/g)	
Fish and seafood	Sea-bass	Eutrophication_potencial (g PO4-eq/g)	0.00025
Fish and seafood	Shrimp	GHG (g CO2-eq/g)	8.74
Fish and seafood	Shrimp	LU (m2/g)	
Fish and seafood	Shrimp	EU (kJ/g)	
Fish and seafood	Shrimp	Acidification_potencial (g SO2-eq/g)	
Fish and seafood	Shrimp	Eutrophication_potencial (g PO4-eq/g)	0.000268
Fish and seafood	Tilapia	GHG (g CO2-eq/g)	16.1
Fish and seafood	Tilapia	LU (m2/g)	
Fish and seafood	Tilapia	EU (kJ/g)	
Fish and seafood	Tilapia	Acidification_potencial (g SO2-eq/g)	
Fish and seafood	Tilapia	Eutrophication_potencial (g PO4-eq/g)	
Fish and seafood	Trout	GHG (g CO2-eq/g)	3.645714286
Fish and seafood	Trout	LU (m2/g)	
Fish and seafood	Trout	EU (kJ/g)	
Fish and seafood	Trout	Acidification_potencial (g SO2-eq/g)	
Fish and seafood	Trout	Eutrophication_potencial (g PO4-eq/g)	0.00004998
Fish and seafood	Char	GHG (g CO2-eq/g)	56.6
Fish and seafood	Char	LU (m2/g)	
Fish and seafood	Char	EU (kJ/g)	
Fish and seafood	Char	Acidification_potencial (g SO2-eq/g)	
Fish and seafood	Char	Eutrophication_potencial (g PO4-eq/g)	0.0000357
Fish and seafood	Turbot	GHG (g CO2-eq/g)	8.155
Fish and seafood	Turbot	LU (m2/g)	
Fish and seafood	Turbot	EU (kJ/g)	

Fish and seafood	Turbot	Acidification_potencial (g SO2-eq/g)	
Fish and seafood	Turbot	Eutrophication_potencial (g PO4-eq/g)	0.000357
Fish and seafood	Cod	GHG (g CO2-eq/g)	4.040065217
Fish and seafood	Cod	LU (m2/g)	
Fish and seafood	Cod	EU (kJ/g)	
Fish and seafood	Cod	Acidification_potencial (g SO2-eq/g)	
Fish and seafood	Cod	Eutrophication_potencial (g PO4-eq/g)	
Fish and seafood	Crab	GHG (g CO2-eq/g)	23.44
Fish and seafood	Crab	LU (m2/g)	
Fish and seafood	Crab	EU (kJ/g)	
Fish and seafood	Crab	Acidification_potencial (g SO2-eq/g)	
Fish and seafood	Crab	Eutrophication_potencial (g PO4-eq/g)	
Fish and seafood	Eel	GHG (g CO2-eq/g)	3.61
Fish and seafood	Eel	LU (m2/g)	
Fish and seafood	Eel	EU (kJ/g)	
Fish and seafood	Eel	Acidification_potencial (g SO2-eq/g)	
Fish and seafood	Eel	Eutrophication_potencial (g PO4-eq/g)	
Fish and seafood	Flat fish	GHG (g CO2-eq/g)	16.03846154
Fish and seafood	Flat fish	LU (m2/g)	
Fish and seafood	Flat fish	EU (kJ/g)	
Fish and seafood	Flat fish	Acidification_potencial (g SO2-eq/g)	
Fish and seafood	Flat fish	Eutrophication_potencial (g PO4-eq/g)	0.00000107
Fish and seafood	Herring	GHG (g CO2-eq/g)	1.3148
Fish and seafood	Herring	LU (m2/g)	
Fish and seafood	Herring	EU (kJ/g)	
Fish and seafood	Herring	Acidification_potencial (g SO2-eq/g)	
Fish and seafood	Herring	Eutrophication_potencial (g PO4-eq/g)	
Fish and seafood	Mackerel	GHG (g CO2-eq/g)	2.628
Fish and seafood	Mackerel	LU (m2/g)	
Fish and seafood	Mackerel	EU (kJ/g)	
Fish and seafood	Mackerel	Acidification_potencial (g SO2-eq/g)	
Fish and seafood	Mackerel	Eutrophication_potencial (g PO4-eq/g)	3.45233E-06
Fish and seafood	Pollock	GHG (g CO2-eq/g)	3.326666667

Fish and seafood	Pollock	LU (m2/g)	
Fish and seafood	Pollock	EU (kJ/g)	
Fish and seafood	Pollock	Acidification_potencial (g SO2-eq/g)	
Fish and seafood	Pollock	Eutrophication_potencial (g PO4-eq/g)	
Fish and seafood	Snapper	GHG (g CO2-eq/g)	5.294
Fish and seafood	Snapper	LU (m2/g)	
Fish and seafood	Snapper	EU (kJ/g)	
Fish and seafood	Snapper	Acidification_potencial (g SO2-eq/g)	
Fish and seafood	Snapper	Eutrophication_potencial (g PO4-eq/g)	
Fish and seafood	Swordfish	GHG (g CO2-eq/g)	14.5
Fish and seafood	Swordfish	LU (m2/g)	
Fish and seafood	Swordfish	EU (kJ/g)	
Fish and seafood	Swordfish	Acidification_potencial (g SO2-eq/g)	
Fish and seafood	Swordfish	Eutrophication_potencial (g PO4-eq/g)	
Fish and seafood	Tuna	GHG (g CO2-eq/g)	9.438571429
Fish and seafood	Tuna	LU (m2/g)	
Fish and seafood	Tuna	EU (kJ/g)	
Fish and seafood	Tuna	Acidification_potencial (g SO2-eq/g)	
Fish and seafood	Tuna	Eutrophication_potencial (g PO4-eq/g)	0.0000119
Fish and seafood	Anglerfish	GHG (g CO2-eq/g)	62.5
Fish and seafood	Anglerfish	LU (m2/g)	
Fish and seafood	Anglerfish	EU (kJ/g)	
Fish and seafood	Anglerfish	Acidification_potencial (g SO2-eq/g)	
Fish and seafood	Anglerfish	Eutrophication_potencial (g PO4-eq/g)	
Fish and seafood	Squid	GHG (g CO2-eq/g)	13.76666667
Fish and seafood	Squid	LU (m2/g)	
Fish and seafood	Squid	EU (kJ/g)	
Fish and seafood	Squid	Acidification_potencial (g SO2-eq/g)	
Fish and seafood	Squid	Eutrophication_potencial (g PO4-eq/g)	
Pork	Pork	GHG (g CO2-eq/g)	6.987946539
Pork	Pork	LU (m2/g)	0.024627889
Pork	Pork	EU (kJ/g)	39.74850289
Pork	Pork	Acidification_potencial (g SO2-eq/g)	0.305239562

Pork	Pork	Eutrophication_potencial (g PO4-eq/g)	0.070722188
Poultry	Poultry	GHG (g CO2-eq/g)	5.898333333
Poultry	Poultry	LU (m2/g)	0.013966667
Poultry	Poultry	EU (kJ/g)	27.8625
Poultry	Poultry	Acidification_potencial (g SO2-eq/g)	0.1733
Poultry	Poultry	Eutrophication_potencial (g PO4-eq/g)	0.051666667
Ruminant Meat	Beef	GHG (g CO2-eq/g)	40.53150685
Ruminant Meat	Beef	LU (m2/g)	0.192843478
Ruminant Meat	Beef	EU (kJ/g)	61.21111111
Ruminant Meat	Beef	Acidification_potencial (g SO2-eq/g)	0.591655385
Ruminant Meat	Beef	Eutrophication_potencial (g PO4-eq/g)	0.323145455
Ruminant Meat	Mutton and Goat	GHG (g CO2-eq/g)	50.41538462
Ruminant Meat	Mutton and Goat	LU (m2/g)	0.184066667
Ruminant Meat	Mutton and Goat	EU (kJ/g)	31.9
Ruminant Meat	Mutton and Goat	Acidification_potencial (g SO2-eq/g)	1.654
Ruminant Meat	Mutton and Goat	Eutrophication_potencial (g PO4-eq/g)	0.6965
	Almond	GHG (g CO2-eq/g)	0.00125
	Almond	LU (m2/g)	
	Almond	EU (kJ/g)	
	Almond	Acidification_potencial (g SO2-eq/g)	
	Almond	Eutrophication_potencial (g PO4-eq/g)	
	Prespa beans	GHG (g CO2-eq/g)	0.23122449
	Prespa beans	LU (m2/g)	0.004744898
	Prespa beans	EU (kJ/g)	1.772188776
	Prespa beans	Acidification_potencial (g SO2-eq/g)	0.011239286
	Prespa beans	Eutrophication_potencial (g PO4-eq/g)	0.001751531
	Soybean	GHG (g CO2-eq/g)	0.210824176
	Soybean	LU (m2/g)	0.003791667
	Soybean	EU (kJ/g)	2.938
	Soybean	Acidification_potencial (g SO2-eq/g)	0.0044
	Soybean	Eutrophication_potencial (g PO4-eq/g)	0.003671429
	Butter	GHG (g CO2-eq/g)	10.86685714
	Butter	LU (m2/g)	0.00952381

Butter	EU (kJ/g)	
Butter	Acidification_potencial (g SO2-eq/g)	0.066230357
Butter	Eutrophication_potencial (g PO4-eq/g)	0.031198214
Olive oil	GHG (g CO2-eq/g)	8.5425
Olive oil	LU (m2/g)	
Olive oil	EU (kJ/g)	
Olive oil	Acidification_potencial (g SO2-eq/g)	
Olive oil	Eutrophication_potencial (g PO4-eq/g)	
Palm oil	GHG (g CO2-eq/g)	2.733333333
Palm oil	LU (m2/g)	
Palm oil	EU (kJ/g)	
Palm oil	Acidification_potencial (g SO2-eq/g)	1.57143E-05
Palm oil	Eutrophication_potencial (g PO4-eq/g)	
Peanut oil	GHG (g CO2-eq/g)	5.007857143
Peanut oil	LU (m2/g)	0.018785714
Peanut oil	EU (kJ/g)	
Peanut oil	Acidification_potencial (g SO2-eq/g)	
Peanut oil	Eutrophication_potencial (g PO4-eq/g)	
Rapeseed oil	GHG (g CO2-eq/g)	6.542142857
Rapeseed oil	LU (m2/g)	0.002357143
Rapeseed oil	EU (kJ/g)	
Rapeseed oil	Acidification_potencial (g SO2-eq/g)	4.28571E-05
Rapeseed oil	Eutrophication_potencial (g PO4-eq/g)	
Sunflower oil	GHG (g CO2-eq/g)	2.705
Sunflower oil	LU (m2/g)	0.011142857
Sunflower oil	EU (kJ/g)	
Sunflower oil	Acidification_potencial (g SO2-eq/g)	
Sunflower oil	Eutrophication_potencial (g PO4-eq/g)	
Table sugar	GHG (g CO2-eq/g)	0.8175
Table sugar	LU (m2/g)	0.00125
Table sugar	EU (kJ/g)	
Table sugar	Acidification_potencial (g SO2-eq/g)	
Table sugar	Eutrophication_potencial (g PO4-eq/g)	

Beer	GHG (g CO2-eq/g)	0.145397985
Beer	LU (m2/g)	
Beer	EU (kJ/g)	
Beer	Acidification_potencial (g SO2-eq/g)	
Beer	Eutrophication_potencial (g PO4-eq/g)	
Wine	GHG (g CO2-eq/g)	1.380650667
Wine	LU (m2/g)	0.0075808
Wine	EU (kJ/g)	0.0000192
Wine	Acidification_potencial (g SO2-eq/g)	0.0000096
Wine	Eutrophication_potencial (g PO4-eq/g)	0.00000896
